# Prophylactic Intra-abdominal Drains in Major Elective Surgeries: A Comprehensive Review

**DOI:** 10.7759/cureus.54056

**Published:** 2024-02-12

**Authors:** Sai Goutham Rekavari, Chanrashekhar Mahakalkar

**Affiliations:** 1 General Surgery, Jawaharlal Nehru Medical College, Datta Meghe Institute of Higher Education & Research, Wardha, IND

**Keywords:** shared decision-making, evidence-based recommendations, clinical practice, postoperative complications, major elective surgeries, prophylactic intra-abdominal drains

## Abstract

This comprehensive review explores the use of prophylactic intra-abdominal drains in major elective surgeries, offering a retrospective analysis of their historical evolution, current evidence, and implications for clinical practice. The definition and rationale for drain placement are elucidated, emphasizing their role in preventing postoperative complications. The review synthesizes conflicting evidence, acknowledging the efficacy of drains in specific surgical contexts while addressing concerns and criticisms regarding associated complications. The implications for clinical practice underscore the importance of a nuanced and individualized approach, incorporating shared decision-making between healthcare providers and patients. Looking ahead, areas for future research are identified, including the refinement of patient selection criteria, determination of optimal timing and duration of drain use, and exploration of innovative alternatives. This review contributes to the ongoing discourse as surgical practices evolve, providing insights that may shape evidence-based recommendations and refine best practices in major elective surgeries.

## Introduction and background

Prophylactic intra-abdominal drains, commonly employed in surgical procedures, refer to the preemptive placement of drainage tubes within the abdominal cavity. These drains are strategically positioned to facilitate the removal of potential accumulations of fluid or air, with the overarching goal of preventing postoperative complications [1]. Incorporating prophylactic intra-abdominal drains in major elective surgeries stems from a multifaceted rationale. Surgical interventions, particularly those categorized as primary and elective, often carry an inherent risk of postoperative complications, such as intra-abdominal fluid collections, hematoma formation, or abscesses. Prophylactic drains are hypothesized to mitigate these risks by promoting the timely evacuation of fluids and preventing their accumulation, thereby contributing to improved patient outcomes [2].

The significance of exploring the role of prophylactic intra-abdominal drains in major elective surgeries lies in its potential impact on patient safety, recovery, and surgical management. As surgical practices continue to evolve, understanding the historical context, current controversies, and emerging trends surrounding the use of these drains becomes pivotal for informed decision-making in the medical community [3]. This review aims to critically examine the utility and effectiveness of prophylactic intra-abdominal drains in the context of major elective surgeries. By synthesizing existing literature, assessing historical perspectives, and analyzing current guidelines, the review intends to provide a comprehensive overview. Specific objectives include evaluating the evidence supporting drain use, exploring controversies and criticisms, summarizing current guidelines, and identifying emerging trends that may shape future surgical practices. Through this exploration, we aim to contribute to the ongoing discourse on optimal perioperative care and patient outcomes.

## Review

Types of major elective surgeries

Categorization of Major Elective Surgeries

Cosmetic surgeries constitute a category of medical interventions to enhance a person's physical appearance. Elective procedures include facelifts, nose jobs (rhinoplasty), breast augmentation, liposuction, and lip augmentation. Individuals often choose cosmetic surgeries to address specific aesthetic concerns, aiming to improve their appearance, foster increased self-confidence, and contribute to overall well-being [4]. Organ transplant surgeries involve the removal of a damaged or dysfunctional organ from a recipient and replacement with a healthy organ from a donor. This category encompasses critical procedures like kidney transplants, liver transplants, and heart transplants. Organ transplant surgeries play a pivotal role in restoring vital organ function, often saving lives and significantly improving the quality of life for recipients [5].

Major abdominal surgeries are characterized by extensive procedures within the abdominal cavity. Examples include bowel resection, gastrectomy, and pancreatectomy. These surgeries involve intricate techniques and are typically necessary for treating conditions affecting abdominal organs, such as tumors or inflammatory diseases [6]. Cardiac surgeries focus on the heart and the cardiovascular system. Procedures like coronary artery bypass grafting, valve replacement, and heart transplants fall under this category. Crucial for addressing structural or functional issues with the heart, cardiac surgeries aim to restore optimal cardiovascular health [5].

Reconstructive surgeries are designed to restore or enhance the function and appearance of a body part affected by congenital anomalies, trauma, or disease. Examples include cleft lip repair, scoliosis surgery, and limb reconstruction. These interventions are vital for improving the overall quality of life and functional abilities of individuals impacted by physical deformities or impairments [6]. Minor surgeries encompass a range of superficial procedures that do not require penetration of a body cavity. Examples include biopsies, repairs of cuts or small wounds, and the removal of warts, benign skin lesions, hemorrhoids, or abscesses. Although less invasive than significant surgeries, minor procedures are essential for diagnosing and treating minor conditions and addressing cosmetic concerns [6].

Overview of Intra-abdominal Complications in Each Surgery Type

An investigation into predictors of major complications following elective abdominal surgery in cancer patients revealed that individuals undergoing abdominal surgery for solid tumors often experience significant postoperative complications. These complications can adversely affect the patient's quality of life, increase care costs, and impact overall survival rates. The study, encompassing patients undergoing colorectal and gynecological procedures, observed a substantial proportion requiring admission to the intensive care unit (ICU) following surgery [7]. In another study, a comparative assessment of surgical outcomes following emergency abdominal exploration for complications of elective surgery and high-risk primary emergencies indicated that patients undergoing complex elective abdominal surgery are appropriately evaluated to identify factors amenable to intervention before the surgery. This preoperative assessment was associated with potentially reducing the risk of postoperative complications. However, the study also underscored the relatively low complication burden in the elective group, juxtaposed with markedly high mortality rates, reflecting the intrinsically high-risk nature of abdominal events [8].

Examining temporal patterns of significant complications after intra-abdominal operations, another study identified diverse major complications occurring at distinct time intervals, ranging from the early to late postoperative periods. Recognizing these patterns was deemed crucial for effective clinical management [9]. A comprehensive review focusing on complications in colorectal surgery identified intraoperative complications, such as bleeding, bowel injury, ureteral lesions, and bladder injuries, primarily attributable to intra-abdominal factors. The review underscored the importance of clearly defining and grading postoperative complications, explicitly emphasizing the Clavien-Dindo Classification's reliability as a quality assessment tool [10]. Furthermore, a study delving into postoperative complications and mortality after major abdominal surgery detailed the incidence of postoperative complications, length of hospital stays, and mortality rates. This study provided valuable insights into the overall occurrence of postoperative complications and mortality among patients undergoing major abdominal surgery [11]. Various factors, including the specific surgical procedure, patient characteristics, and the timing of complications, influence the incidence of intra-abdominal complications in major elective surgeries. A thorough understanding of these factors is imperative for the comprehensive assessment and effective management of risks associated with major elective abdominal surgeries.

Current Practices Regarding Prophylactic Drains in Specific Surgeries

Colorectal surgery: Including prophylactic drains in colorectal surgery has sparked an ongoing debate over the past few decades. Recent studies have challenged the conventional belief that intraperitoneal drains prevent anastomotic leaks. Instead, current evidence suggests that these drains do not sufficiently mitigate the risk of anastomotic leaks. The contemporary standard of care has shifted toward percutaneous drain placement, recognized as a more practical approach for managing intra-abdominal collections. Even in selected anastomotic leaks in stable patients, percutaneous drains have emerged as a viable management strategy [12].

Liver resections: The routine practice of prophylactic drain insertion in abdominal surgery has faced scrutiny, particularly concerning its efficacy in major liver resections. Contrary to previous assumptions, some studies indicate that drains do not significantly reduce bile leakage, bleeding, or the necessity for additional drain insertions following liver resections. The decision to incorporate or omit drains in such cases is now acknowledged as a nuanced choice, dependent on individualized considerations and the surgeon's expertise. This reflects a paradigm shift in evaluating the role of prophylactic drains in significant liver resections [13].

Gastrointestinal surgeries: Randomized studies have failed to provide a clear advantage in favor of routine prophylactic drainage after gastrectomy in gastrointestinal surgeries. Similarly, a meta-analysis focusing on short-term outcomes following distal pancreatectomy revealed that intra-abdominal drainage did not yield improvements. The use of prophylactic drains in specific gastrointestinal surgeries has become a subject of ongoing debate. The decision to employ or forego drains should be rooted in individualized choices guided by the surgeon's expertise. Recognizing the complexities involved, it is imperative to carefully weigh the benefits and risks associated with drain usage for each patient and specific surgical procedure [1,12,14].

Mechanism and function of intra-abdominal drains

Drain Placement and Function

Ensuring the appropriate placement of surgical drains is paramount to optimizing their functionality and mitigating potential complications. The technique for placement varies depending on the specific type of drain employed. For instance, when positioning an active suction drain within the peritoneal cavity, careful consideration should be given to selecting an exit point away from the primary incision. This strategic placement aims to reduce the risk of complications such as infection and dehiscence. The insertion process demands strict adherence to the aseptic technique, and determining the exit location should follow general principles, such as avoiding the drain's exit through the leading incision site to prevent bacterial entry along the incision length [15].

Surgical drains serve various purposes, encompassing the drainage of potential spaces, monitoring outputs, and detecting bleeds or leaks. These drains are categorized based on their function as open or closed and passive or active. Proper management and vigilant monitoring of the drain's output are imperative. The indication for its insertion should inform the decision to remove the drain and align with clear instructions [16,17]. Recognizing that the placement of surgical drains is a critical determinant of their efficacy, it is crucial to execute this process with precision to maximize effectiveness and minimize the potential for complications. Considerations such as the type of drain, its intended function, and the patient's overall condition should all be considered when placing and managing surgical drains [15,17].

Drainage Mechanisms and Fluid Dynamics

Fluid dynamics: Drains are strategically placed in anatomical spaces that have the propensity to accumulate fluids, including but not limited to the subhepatic, right subphrenic, left subphrenic, and peripancreatic spaces. These spaces are commonly associated with surgical interventions in the abdominal cavity. The utilization of drains serves multiple purposes, encompassing diagnostic, prophylactic, and therapeutic objectives. By targeting these specific spaces, surgeons aim to prevent the accumulation of fluids that could potentially lead to complications or hinder the postoperative healing process [18].

Drainage mechanisms: Drains operate through either open-circuit or closed-circuit systems. The choice between these mechanisms holds significant implications for postoperative outcomes. Open-circuit drains are linked to a higher incidence of abdominal complications, primarily stemming from intra-abdominal infections, in contrast to closed-circuit drains, which are generally associated with a lower risk of complications. The preference for closed-circuit drains is evident due to their efficacy in minimizing potential adverse events in most cases [19].

Drain route: The routes through which drains are placed are diverse, with considerations to avoid traversing intra-abdominal organs or the thorax in some instances. Percutaneous drain routes involving the penetration of intra-abdominal organs or the thorax are also employed based on the specific surgical procedure and the surgeon's expertise. The selection of the drain route is a critical decision influenced by the nature of the surgery and the surgeon's understanding of the anatomical intricacies involved [12].

Early detection of complications: Prophylactic drainage facilitates the early detection of complications, such as anastomotic leakage. This proactive approach allows healthcare professionals to identify potential issues swiftly and intervene promptly, potentially improving patient outcomes. It is noteworthy, however, that there is no unanimous consensus regarding the routine use of drains, and their incorporation is not endorsed in the "Enhanced Recovery After Surgery" (ERAS) protocols. The decision to use intra-abdominal drains hinges on the need to evacuate harmful or potentially infected fluids and the imperative of early complication detection. The surgeon's expertise and the specifics of the surgical procedure play pivotal roles in determining the optimal type, route, and mechanism of the drain employed [12].

Impact on Postoperative Outcomes

The influence of intra-abdominal drains on postoperative outcomes remains a topic of considerable debate. Although specific studies indicate that the preventive insertion of drains does not lead to a reduction in complications, such as bile leakage, bleeding, or the necessity for additional drain insertions [19], contrasting findings from other studies report elevated rates of leaks, prolonged hospital stays, and increased costs associated with this practice [13]. In the context of liver transplantation, the use of abdominal drains is commonplace, yet their definitive impact on postoperative complications remains uncertain. Some studies argue that prophylactic drains fail to diminish complications like bile leakage, bleeding, or the need for additional drain insertions [19]. Conversely, additional research contends that implementing prophylactic drains correlates with heightened leak rates, prolonged hospital stays, and increased costs [13].

The role of prophylactic abdominal drainage in pancreatic surgery has stirred controversy regarding its efficacy in reducing postoperative complications [20]. While specific studies propose that early drain removal may lead to a decrease in intra-abdominal infection rates, morbidity, and length of hospital stay compared to late drain removal [20], the available evidence lacks consensus. The impact of drain use on postoperative outcomes is nuanced and varies depending on the specific surgical procedure and patient population. The overall influence of intra-abdominal drains on postoperative outcomes has yet to be definitively established, necessitating a case-by-case evaluation and reliance on the surgeon's expertise in decision-making. While some studies advocate for the benefits of drain usage, others caution that prophylactic insertion may not necessarily reduce complications and could potentially exacerbate specific outcomes [13].

Efficacy and benefits

Review of Studies Supporting the Use of Prophylactic Drains

Numerous studies have assessed the efficacy and advantages of employing prophylactic intra-abdominal drains in major elective surgeries, leading to a divergence in perspectives. A non-randomized comparative study conducted in a teaching hospital revealed that placing prophylactic drains after gastrointestinal surgery did not confer additional benefits. Instead, it was associated with increased operative duration, prolonged hospital stays, and higher surgical site infection (SSI) rates [21]. In a broader context, a systematic review and meta-analysis focusing on the evidence-based value of prophylactic drainage in gastrointestinal surgery concluded that many gastrointestinal operations can be safely performed without resorting to prophylactic drainage. Specifically, the review suggested the omission of drains after hepatic, colonic, or rectal resection with primary anastomosis and appendectomy for any stage of appendicitis [22].

Examining the prophylactic use of drains in colorectal surgery, a systematic review by the Cochrane Collaboration found that intraperitoneal drains did not adequately prevent anastomotic leaks [12]. An umbrella review encompassing systematic reviews and meta-analyses on the safety and efficacy of prophylactic drainage after intra-abdominal surgeries concluded that data from randomized studies failed to demonstrate any advantage in routine prophylactic drainage after gastrectomy [14]. Similarly, a study investigating the need for prophylactic intra-abdominal drainage in distal pancreatectomy found that such drainage did not yield improved outcomes [1].

While specific studies advocate for the utility of prophylactic intra-abdominal drains, others question their necessity, highlighting the variability in findings across different surgical procedures. The decision to employ or forgo drains should be grounded in individualized choices and the surgeon's expertise, underscoring the importance of a personalized approach to drain usage in major elective surgeries [22].

Reduction in Postoperative Complications

The utilization of prophylactic intra-abdominal drains in major elective surgeries has been recognized for its potential to prevent or manage postoperative complications, including intra-abdominal abscesses and pancreatic fistulas [20,23]. However, the benefits of drains must be weighed against the potential occurrence of rare adverse events, such as bowel perforation, hernias, and bleeding [20]. Several studies have suggested that routine prophylactic abdominal drains may not significantly reduce 30-day mortality. Still, low-certainty evidence indicates a potential reduction in mortality at 90 days [20]. Early removal of drains has been associated with a decrease in the rate of intra-abdominal infections, morbidity, and the length of hospital stay [20]. Moreover, the duration of drain insertion has been identified as a significant risk factor for intra-abdominal infections, emphasizing that early removal of drains independently contributes to reducing the incidence of such infections [23].

In the specific context of significant liver resections, prophylactic drains have shown limited effectiveness in reducing bile leakage, bleeding, or the need for additional drain insertions. Conversely, their use has been linked to higher rates of leaks, prolonged hospital stays, and increased costs [13]. In addition, certain studies have indicated that drains do not significantly aid in diagnosing postoperative bleeding earlier or improving treatment outcomes [13]. While prophylactic intra-abdominal drains may hold the potential to prevent specific postoperative complications, their use is also associated with rare adverse events. The decision to incorporate drains should be meticulously considered, considering the specific surgical context and individual patient factors. Early removal of drains is a beneficial strategy in reducing the incidence of intra-abdominal infections and overall morbidity. Careful evaluation and personalized decision-making are crucial in optimizing the outcomes associated with prophylactic intra-abdominal drain usage in major elective surgeries.

Patient Outcomes and Satisfaction

Malignant ascites: A systematic review comprising 32 original articles delved into the efficacy of permanent drain insertion in patients with malignant ascites. The findings indicated that those selected for drain insertion experienced improvements in symptom control following ascites drainage. However, it is noteworthy that the primary focus of the articles centered on procedural safety issues, with a limited number of studies reporting patient-reported outcomes (PRO) [24].

Elective laparoscopic cholecystectomy: A systematic review and meta-analysis explored factors influencing the length of stay (LOS) in elective laparoscopic cholecystectomy. Interestingly, the review found that prophylactic drain insertion was not associated with an increased LOS. However, it is crucial to note that the studies examined lacked sufficient power for meta-analysis, highlighting a potential limitation in the available evidence [25].

Colorectal surgery: An internationally matched, prospective cohort study scrutinized drain insertion in colorectal surgery patients, revealing that those who received drains often had higher ASA grades and underwent open surgical approaches more frequently. However, upon adjusting for confounding by indication, the study concluded that drain insertion was not associated with reduced rates of postoperative complications [26].

Appendectomy for complicated appendicitis: A meta-analysis investigated the effects of prophylactic abdominal drainage on postoperative complications in patients with complicated appendicitis. The analysis revealed that drains did not significantly reduce the risk of postoperative complications, providing insight into the limited impact of drain insertion in this specific surgical context [27].

Cytoreductive surgery: A systematic review and meta-analysis of randomized controlled trials focused on prophylactic intra-abdominal drainage following colorectal anastomoses in cytoreductive surgery. The comprehensive analysis indicated that drain insertion did not significantly improve outcomes, shedding light on the nuanced role of drains in this specific surgical setting [28].

Controversies and criticisms

Contradictory Findings in the Literature

Pancreatic surgery: The role of prophylactic abdominal drainage to mitigate postoperative complications after pancreatic surgery is controversial, as a Cochrane review indicates [20]. The review highlighted substantial uncertainty in the evidence regarding the impact of drain use on the intra-abdominal infection rate and the necessity for additional radiological interventions for postoperative complications. The discussion surrounding the effectiveness of prophylactic drainage in enhancing outcomes after pancreatic surgery remains inconclusive [20].

Major liver resections: A systematic review and meta-analysis of randomized controlled trials focused on major liver resections revealed that prophylactic intra-abdominal drainage did not contribute to a reduction in bile leakage, bleeding, or the requirement for additional drain insertion. Notably, most of these studies predominantly involved minor liver resections, leaving the role of abdominal drain insertion after major liver resections in a state of controversy. The effectiveness of prophylactic drainage in the context of significant liver resections remains uncertain [13].

Colorectal surgery: In the realm of colorectal surgery, several studies have challenged the efficacy of intraperitoneal drains in preventing anastomotic leaks. Consequently, due to these findings, prophylactic drainage of the peritoneal cavity has witnessed a decline in popularity. Nevertheless, percutaneous drain placement has emerged as the standard of care for patients with intra-abdominal collections, presenting a shift in the approach to managing postoperative complications in colorectal surgery [12].

Gastrointestinal surgeries: Randomized studies fail to provide a clear advantage in favor of routine prophylactic drainage after gastrectomy in gastrointestinal surgeries. The literature presents conflicting findings concerning prophylactic intra-abdominal drains in major elective surgeries. While some studies suggest that drains may not be effective in preventing complications, others argue that they can enhance early detection of complications and prevent collections. The decision to employ or forego drains is based on individualized choices and the surgeon's expertise, underlining the complex and personalized nature of this decision-making process [1,14,20].

Complications Associated With Intra-abdominal Drains

Complications linked with the utilization of intra-abdominal drains encompass infection, thoracic issues related to pain leading to microatelectasis, and the potential to exacerbate intra-abdominal hemorrhage or hematoma [29]. The employment of prophylactic abdominal drainage may also carry rare adverse events, including but not limited to bowel perforation, hernias, and bleeding [12,20]. Furthermore, the routine implementation of prophylactic abdominal drains might be associated with an extended hospital stay [20]. Other potential complications involve issues with the operative wound (such as abscess, disruption, or incisional hernia), pulmonary concerns like microatelectasis, intestinal obstructions, ulcerations leading to fistulae, hemorrhages, infections along the drainage tract, challenges in removal, intra-abdominal retention, and disruptions in the incision site [29]. Nonetheless, the decision to employ or forgo drains should be rooted in individualized choices and the expertise of the surgeon, recognizing the need for a personalized approach in this decision-making process [29].

Disagreements Among Medical Professionals

Efficacy: The effectiveness of prophylactic drain insertion in major elective surgeries remains to be determined. Some studies indicate that the insertion of prophylactic drains may not significantly reduce issues like bile leakage, bleeding, or the necessity for additional drain insertion. However, contrasting findings in other studies suggest that prophylactic drains might play a role in decreasing the occurrence of postoperative complications. The variability in results underscores the complexity of determining the overall efficacy of prophylactic drains in these surgical contexts [12,13].

Length of hospital stay: Drains have been associated with an extended length of hospital stay, posing a concern for both patients and healthcare providers. Prolonged hospital stays increase the financial burden on patients and raise potential complications associated with extended hospitalization. This emphasizes the need to carefully assess the trade-offs between the perceived benefits of drain usage and the implications of an extended hospital stay [20].

Adverse events: Prophylactic abdominal drainage may be linked to rare adverse events, including bowel perforation, hernia, and bleeding. These complications have the potential to necessitate additional medical interventions and contribute to prolonged hospital stays. Considering potential adverse events becomes critical in weighing the overall risk-benefit profile of prophylactic drain insertion in major elective surgeries [20].

Cost: Some studies have indicated that using prophylactic drains is associated with higher rates of leaks, prolonged hospital stays, and increased overall costs. This raises significant questions about the cost-effectiveness of employing drains in major elective surgeries. Evaluating the financial implications becomes pivotal in determining the utility and value of prophylactic intra-abdominal drains in healthcare resource utilization [13].

Individualized choices: The decision to utilize or abstain from prophylactic drains should be grounded in individualized choices and the surgeon's expertise. This underscores the importance of tailoring the approach to each patient's unique needs and considering specific risk factors when deciding on the appropriateness of prophylactic intra-abdominal drains. The individualized approach acknowledges the inherent variability among patients and the need for personalized decision-making in surgical management [20].

Current guidelines and recommendations

Overview of Major Surgical Societies’ Guidelines

Various surgical societies have issued guidelines and recommendations covering diverse facets of surgical care. In 2019, the American Society of Hematology (ASH) released guidelines addressing the prevention of venous thromboembolism in surgically hospitalized patients. These guidelines span recommendations for various surgical categories, encompassing major surgery in general, orthopedic surgery, major general surgery, major neurosurgical procedures, urological surgery, cardiac surgery, major vascular surgery, major trauma, and significant gynecological surgery [30]. The Surgical Infection Society has also provided guidelines about antimicrobial therapy for intra-abdominal infections and managing intra-abdominal infections [31].

A comprehensive study conducted in 2019 systematically reviewed the Choosing Wisely recommendations by surgical societies. The findings revealed active participation by numerous major surgical societies in the Choosing Wisely campaign. This engagement contributed to the campaign's goals by endorsing evidence-based de-implementation strategies [32]. In response to the challenges posed by the COVID-19 pandemic, several surgical societies have issued guidelines and recommendations for surgical practices. These encompass guidance on the triage and prioritization of cases, ensuring patient and staff safety, and offering practical insights into the management of emergency surgery during the pandemic [33].

Consensus Statements on Prophylactic Intra-abdominal Drains

The utilization of prophylactic intra-abdominal drains in major elective surgeries remains a contentious issue marked by a need for more consensus. Recent trends indicate a decline in the popularity of prophylactic drainage of the peritoneal cavity, driven by multiple studies revealing that intraperitoneal drains fail to prevent anastomotic leaks effectively [12]. This debate has been particularly persistent in colorectal surgery, with ongoing discussions spanning several decades and recent research reinforcing the argument that intraperitoneal drains do not adequately prevent anastomotic leaks [20]. A systematic review and meta-analysis investigating the efficacy of prophylactic abdominal drainage in significant liver resections indicated that drains did not mitigate issues, such as bile leakage, bleeding, or additional drain insertions [12]. However, counterintuitive findings emerged from other studies, associating prophylactic drains with heightened risks of leaks, prolonged hospital stays, and increased overall costs [12]. This conflicting evidence underscores the complexity of evaluating prophylactic drains' overall impact and cost-effectiveness in major elective surgeries.

In gastrointestinal surgeries, randomized studies fail to provide a clear advantage in supporting routine prophylactic drainage after gastrectomy [1]. Similarly, for distal pancreatectomy, a meta-analysis focusing on short-term outcomes concluded that intra-abdominal drainage did not yield significant improvements [14]. The cumulative data from randomized studies fail to establish a consistent advantage in favor of routine prophylactic drainage in major elective surgeries. Amid this lack of consensus, it is emphasized that the decision to employ or forgo drains should be grounded in individualized choices and the surgeon's expertise [20]. Acknowledging the variability in outcomes and the nuanced nature of surgical decisions, a personalized approach is deemed essential in determining the appropriateness of prophylactic intra-abdominal drains for each patient undergoing major elective surgery.

Recent Updates and Changes in Recommendations

Recent updates and changes in recommendations across various fields reflect the dynamic nature of evolving knowledge and guidelines. In advertising ethics, the Federal Trade Commission (FTC) took a significant step in June 2023 by announcing updated advertising guides. These revisions addressed deceptive reviews and endorsements, with specific guidance tailored for influencers. The guidelines clarified the timing and manner in which influencers should disclose material connections, focusing on enhancing transparency in online reviews [34]. The COVID-19 Treatment Guidelines have been subject to regular updates since their initiation in 2020. The latest significant revisions, implemented on December 20, 2023, introduced new recommendations for managing COVID-19 and included updates to existing guideline sections. This reflects the ongoing efforts to integrate the latest scientific insights and medical advancements into the guidance for addressing the complexities of the COVID-19 pandemic [35].

Similarly, the Centers for Disease Control and Prevention (CDC) have been proactive in regularly updating their guidelines related to COVID-19. These updates encompass various aspects, including guidelines for healthcare facilities, vaccination requirements for international travelers, and revised guidance for fully vaccinated individuals. These guidelines' adaptability reflects the pandemic's dynamic nature and the need for timely adjustments based on emerging data and evolving circumstances [36]. Recent updates in guidelines have been observed within the domain of primary care, particularly in areas, such as primary spontaneous pneumothorax and evaluations of the efficacy and side effects of medications. These updates underline the commitment to refining and enhancing clinical practices to align with the latest evidence and best practices [35]. Lastly, UpToDate, a reputable medical resource, consistently publishes practice-changing updates. These updates highlight specific new recommendations and alterations that may prompt a shift in usual clinical practices. By regularly disseminating such information, UpToDate informs healthcare professionals about the latest developments that may impact their approach to patient care [37].

Emerging trends and innovations

Advances in Surgical Techniques Affecting Drain Use

Soft-tissue tumor (STT) surgery: Investigations into using surgical drains in STT surgery have yielded insights. Some studies suggest that the placement of a surgical drain does not significantly impact the rate of seroma formation following STT resection [38]. This underscores the nuanced considerations in drain usage in soft-tissue tumor surgeries, where the balance between potential benefits and outcomes remains a subject of ongoing exploration.

Colorectal surgery: In the realm of colorectal surgery, there has been a notable shift in the popularity of prophylactic drainage of the peritoneal cavity. Recent studies have demonstrated that intraperitoneal drains may not effectively prevent complications, leading to a decline in their widespread use. The decision to employ drains or not is increasingly recognized as an individualized choice, contingent upon the surgeon's personalized assessment of the patient's needs and potential benefits [12]. This reflects a trend toward tailoring interventions based on specific clinical contexts.

Percutaneous drainage: Percutaneous drain placement has emerged as the standard for managing intra-abdominal leaks, pivotal in the therapeutic arsenal for various disease processes [12]. This signifies a paradigm shift toward less invasive and more targeted approaches in managing complications, offering patients a potentially more favorable and efficient course of treatment.

Drain fixation: Advancements in surgical drain fixation techniques have addressed limitations associated with existing methods, such as issues related to loosening and skin problems [39]. These innovative approaches indicate a commitment to enhancing the practical aspects of drain management, aiming to improve the overall efficacy and comfort for patients undergoing surgical procedures that necessitate drain placement.

Patient-performed drain removal: Recent research challenges the traditional notion that surgical drains are a relative contraindication to telemedicine-based postoperative care. Studies have demonstrated that patient-performed at-home surgical drain removal can be safe and feasible, showcasing a patient-centered approach to postoperative care [40]. This shift in perspective aligns with the broader trend in healthcare toward empowering patients and exploring novel avenues for optimizing recovery beyond traditional in-hospital settings.

Exploration of Alternative Prophylactic Measures

Alternative prophylactic measures for intra-abdominal drains have been explored across diverse medical disciplines. In the dental field, the recommendation of antibiotic prophylaxis before dental procedures for individuals with specific heart conditions or joint replacements has been considered [41]. However, the potential for adverse reactions to antibiotics generally outweighs the perceived benefits, and the emergence of drug-resistant bacteria poses a significant concern [41]. Within the domain of cardiology, preventive measures for infective endocarditis (IE) encompass the use of prophylactic antibiotics, closure of patent ductus arteriosus or ventricular septal defects, and timely treatment of infections [42-44]. Nevertheless, the utilization of prophylactic antibiotics has been associated with adverse effects, and their application beyond recommended clinical contexts is cautioned against [42]. In gastrointestinal surgeries, a meta-analysis has indicated that prophylactic drainage does not yield improved outcomes following procedures, such as distal pancreatectomy, appendectomy, or gastrectomy [14]. Despite exploring alternative prophylactic measures for intra-abdominal drains in various medical fields, adopting prophylactic antibiotics and defect closure presents its own limitations and associated risks. The decision to employ or forgo prophylactic measures should be grounded in individualized choices, considering the unique circumstances of each case and relying on the physician's expertise.

Future Directions in Research and Practice

Influence of training and peer learning: Future research endeavors could investigate the relative impact of training programs and peer learning experiences on the perspectives and practices of early career researchers concerning the utilization of prophylactic drains in abdominal surgeries [45]. Understanding the interplay between formal training and collaborative learning within research communities can contribute to shaping more effective and informed approaches.

Public involvement in research and development (R&D): There is a critical need to comprehend the implications of involving the public in the R&D process and how much involvement influences research practices. This entails examining how principal investigators (PIs) perceive and integrate public involvement, understanding associated costs, and exploring diverse models of public engagement in the context of prophylactic intra-abdominal drain research [45]. Investigating these dynamics can enhance the transparency and inclusivity of the research process.

Methodological lessons and ethnographic approaches: Research efforts should focus on elucidating the impact of the research process itself on the perspectives and practices of stakeholders involved in prophylactic drain studies. Employing more profound ethnographic approaches, especially by sampling skeptical principal investigators, could offer valuable insights into the intricacies of research dynamics [45]. This approach may uncover implicit factors influencing research outcomes and perceptions.

Addressing research limitations: Future research initiatives should strive to overcome the limitations inherent in existing studies on prophylactic intra-abdominal drains. This involves a critical examination and testing of theoretical models and the identification and proposition of new avenues for research based on the lessons learned from prior studies [46]. This iterative approach can contribute to refining research methodologies and expanding the knowledge base.

Comparative research: There is a necessity for conducting direct and equitable comparisons of different methods for managing intra-abdominal drainage. This includes evaluating standard drainage protocols (SDP), directed drainage protocols (DDP), and multi-criteria analysis (MCA) in a comprehensive manner [47]. Comparative research can elucidate the strengths and limitations of each approach, guiding the development of more effective drainage strategies.

Global trends and practices: Investigating current and anticipated future trends in research and clinical practices on a global scale can provide valuable insights into the evolving approaches to prophylactic intra-abdominal drain usage in major elective surgeries [48]. Understanding global variations and emerging practices can inform best practices and contribute to a more nuanced understanding of regional disparities.

Integration of theory, research, practice, and policy: Future research endeavors should aspire to integrate theoretical advancements, empirical research findings, clinical practices, and policy implications. This holistic approach ensures a comprehensive understanding and application of evidence-based approaches using prophylactic drains in major elective surgeries [49]. Bridging these elements can foster a more cohesive and impactful translational pathway from research to practical implementation and policy formulation.

## Conclusions

This comprehensive review sheds light on the nuanced landscape surrounding prophylactic intra-abdominal drains in major elective surgeries. A spectrum of key findings has emerged by tracing the historical evolution of this practice and synthesizing diverse perspectives from contemporary literature. While specific surgical contexts showcase the efficacy of drains in reducing postoperative complications, the review has also navigated through conflicting evidence and critical voices highlighting potential drawbacks and complications associated with routine drain placement. The implications for clinical practice are substantial, urging surgeons and healthcare practitioners to adopt a more individualized approach, considering the specific characteristics of each surgery and engaging in shared decision-making with patients. As we move forward, areas for future research become evident, including the need to refine patient selection criteria, determine optimal timing and duration of drain use, and explore innovative approaches to minimize complications. In addition, investigating economic implications and comparing outcomes across diverse surgical settings will contribute to the evolution of evidence-based recommendations and best practices in major elective surgeries.
